# In-vitro evaluation of selected native *Trichoderma* species for management of *Pyricularia oryzae*

**DOI:** 10.3389/fmicb.2026.1830098

**Published:** 2026-05-29

**Authors:** Nyamasija Francis Nyakeko, Richard R. Madege, Newton L. Kilasi

**Affiliations:** 1 Department of Agricultural Sciences, Mwalimu Nyerere University of Agriculture and Technology, Chuo Kikuu, Butiama, Tanzania; 2 Department of Crop Science and Horticulture, Sokoine University of Agriculture, Chuo Kikuu, Morogoro, Tanzania; 3 Institute of Pest Management, Sokoine University of Agriculture, Chuo Kikuu, Morogoro, Tanzania

**Keywords:** antagonism, biological control, *P. oryzae*, rice blast disease, native *Trichoderma* spp.

## Abstract

Rice blast, caused by *Pyricularia oryzae*, is one of the most destructive fungal diseases limiting rice productivity worldwide. Species of *Trichoderma* are widely recognized as effective biological control agents against fungal pathogens. This study aimed to evaluate the *in vitro* antagonistic efficacy of native *Trichoderma* spp. isolates collected from Tanzanian lowland rice agroecosystems against *P. oryzae*. A total of 47 native *Trichoderma* spp. isolates were evaluated for antagonistic activity against *P. oryzae.* Direct antagonism was evaluated using dual-culture and slide-culture bioassays and indirect antagonism was evaluated using inversion and culture filtrate methods. These assays measured inhibition of pathogen mycelial growth and sporulation to determine the effectiveness of the isolates. *Trichoderma* isolates significantly inhibited *P. oryzae* across all assays. Dual culture reduced mycelial growth by 63–83% (*p* < 0.001), while non-volatile metabolites caused 57–83% inhibition (*p* < 0.001). VOCs suppressed pathogen growth by 43–75% (*p* < 0.001) and reduced sporulation by up to 90% (*p* < 0.001). Microscopy confirmed mycoparasitism (hyphal coiling, adhesion, penetration), with antibiosis and overgrowth covering 75–100% of colonies. Indigenous isolates outperformed commercial *T. harzianum*, with *T. virens* (KT8-4, NH7-3), *T. harzianum* (NH3-3), *T. pleuroti* (KT8-1), and *T. paraviridescens* (KT7-1) most effective. The strong antagonistic activity of the native *Trichoderma* isolates against *P. oryzae* supports previous reports on their biocontrol potential. These findings highlight their promise as eco-friendly alternatives to chemical fungicides and provide a benchmark for research on identifying active metabolites and validating the most effective isolates under greenhouse and field conditions.

## 1 Introduction

Rice (*Oryza sativa* L.) is the world’s second most important staple crop and plays a critical role in global food security (Ansari et al., 2020). In Tanzania, rice is the second most widely produced food and commercial crop after maize (*Zea mays* L.), with an annual production of approximately 2.2 million metric tons (Msafiri, 2021). It is cultivated on about 995,729 hectares of arable land (FAOSTAT, 2023) and contributes significantly to Tanzania’s national food security, household income, foreign exchange earnings, and gross domestic product (GDP) (Msafiri, 2021).

Rice productivity in Tanzania has increased from 1.51 t ha^−1^ in 1961 to 2.81 t ha^−1^ in 2021 (FAOSTAT, 2023); however, yields remain well below the global potential of 2–5 t ha^−1^. This yield gap is largely attributed to biotic and abiotic stresses. Rice production is severely constrained by diseases such as rice blast, brown spot, bacterial leaf blight and leaf streak, sheath blight, sheath rot, Fusarium wilt, stem rot, Tungro virus, false smut, and post-harvest diseases (Naresh Kumar and Sakthivel, 2025; Simkhada and Thapa, 2022), which collectively account for yield losses of up to 31% worldwide (Song et al., 2025).

Among these diseases, rice blast caused by the filamentous ascomycete fungus *P. oryzae* Cavara is the most destructive disease, occurring in over 85 rice-growing countries and causing yield losses ranging from 30 to 70% (Al Mamun et al., 2026). Severe outbreaks in Africa have resulted in yield losses of up to 100%, particularly in Ghana and parts of Gambia (Mutiga et al., 2021). In Tanzania and other East African countries, blast disease is estimated to cause yield losses of 30–40% (Chuwa, 2016; Kihoro et al., 2013). The pathogen infects rice plants at all growth stages, and severe infection during reproductive and maturity stages can result in total crop failure (Al Mamun et al., 2026). Typical symptoms include spindle-shaped lesions with gray to whitish centers and brown margins that may coalesce under favorable conditions, leading to extensive leaf necrosis.

Several management strategies have been employed to control rice blast, including the use of resistant varieties, fungicides, and cultural practices (Khadka et al., 2025). Resistance breeding is often short-lived due to the rapid evolution of new *P. oryzae* races within 2–3 years (Hashim, 2020). However, recent advances have identified a durable broad-spectrum resistance gene (Pedrozo et al., 2025). Fungicides such as tricyclazole, hexaconazole, and mancozeb are effective, but their excessive and indiscriminate use has led to environmental contamination, human health risks, and the emergence of fungicide-resistant pathogen populations (Mutiga et al., 2021).

Biological control agents (BCAs) offer an environmentally sustainable alternative for managing rice blast. Among them, *Trichoderma* spp. have attracted considerable attention due to their multiple antagonistic mechanisms, including competition for nutrients and space, mycoparasitism, antibiosis, induction of plant defense responses, and plant growth promotion (Prismantoro et al., 2024). More than 25 *Trichoderma* species have been reported to suppress phytopathogens, including *P. oryzae* (Tyśkiewicz et al., 2022). In Tanzania, commercial *T. asperellum* has been reported to reduce rice blast incidence by up to 70% (Hashim, 2020). However, information on the biocontrol potential of native *Trichoderma* isolates remains limited. Therefore, this study aimed to evaluate the antagonistic efficacy of *Trichoderma* species isolated from Tanzanian rice agroecosystems against *P. oryzae in vitro* using direct and indirect antagonism mechanisms.

## 2 Methodology

### 2.1 Isolation of pathogenic fungi

Isolation of *P. oryzae* was done as described by Mathur and Kongsdal (2003). The leaves of rice plant samples showing typical blast symptoms were collected from the field and brought to the plant pathology laboratory at Sokoine University of Agriculture, where they were washed repeatedly with tap water. Small pieces (1 cm) of the diseased portion were cut using a sterilized blade; each piece had some healthy parts as well. The samples were surface sterilized in 1% sodium hypochlorite solution for 1 min, 70% alcohol for 1 min, then rinsed with distilled water three times, placed on a slide, mounted on moist blotting paper, and then incubated at 27 °C.

After 48–72 h. of incubation, spores were observed under a light microscope. The single spore method was used for purification (Hussin et al., 2020), where a single conidium was identified from the sporulating lesions using a stereomicroscope and aseptically transferred to a petri dish (PDA media) and incubated for 7 days to obtain a *P. oryzae* pure culture. A pure culture was assessed for its morphological features, and thereafter, a 7-day-old inoculum was transferred to a fresh rice bran yeast agar (RBYA) plate for sporulation.

### 2.2 *Trichoderma* species

Forty-seven *Trichoderma* isolates (corresponding to *T. harzianum, T. viride, T. longibrachiatum, T. erinaceum, T. koningiopsis, T. atroviride, T. paraviridescens, T. pleuroti,* and *T. asperellum*) isolated from lowland rice agroecosystems in the six agroecological zones of Tanzania were used in this experiment. The spp. were isolated at the Sokoine University of Agriculture plant pathology laboratory Tanzania. This research study focused on isolates from lowland rice agroecosystems, as this system accounts for nearly 80% of Tanzania’s total rice production, underscoring its role in national food security. The areas covering lowland rice agro-ecosystem function as epidemiological hotspots for fungal pathogens, including *P. oryzae*, thereby providing suitable conditions for the co-occurrence and enrichment of natural enemies, such as *Trichoderma* spp.

### 2.3 Evaluation of *Trichoderma* spp. antagonism efficiency against *P. oryzae*

*Trichoderma* spp. antagonism against *P. oryzae* was evaluated using a series of direct and indirect conformation bioassays as described by Kim and Vujanovic (2018); Mokhtari et al. (2018); Singh and Singh (2019); Sriwati et al. (2019) and Steyaert et al. Direct antagonism was evaluated using dual-culture and slide-culture bioassays, whereas indirect antagonism was evaluated using inversion and culture filtrate methods.

#### 2.3.1 Activity 1: assessment of antifungal effects of *Trichoderma* spp. volatile organic metabolites (VOCs) on *P. oryzae* growth and sporulation

To assess *P. oryzae* growth and sporulation inhibition, the inversion method as described by Steyaert et al. was used. A 5 mm agar plug (7 days old) of both fungi (pathogen and endophyte) was inoculated into different petri dishes containing rice bran yeast agar (RBYA). Before dual plating, the *P. oryzae* inoculum was incubated for 72 h to allow *P. oryzae* to grow since it had a lower growth rate. Initial radial growth of *P. oryzae* was recorded. Then the lids of both petri plates were removed, and the plates were overlaid, with *Trichoderma* spp. at the bottom and *P. oryzae* at the top, and sealed together with adhesive tape.

The negative control plate was inoculated with *P. oryzae* without *Trichoderma* isolates, and the positive control was incubated with commercial *T. harzianum* strain. The treatments were incubated at 28 °C for 7 days, in a completely randomized design with four replications. Mycelial radial growth of the pathogen was recorded every 24 h for 7 days of co-incubation. At the end of the incubation period, the percentage of mycelial growth inhibition was determined using the equation given by Edington et al. (1971).

$$\text{Mycelial growth Inihibition}\;(\mathrm{GI})\%=\frac{C-T}{C\;}\times 100\;$$ (1)

Where *C* is the radial mycelia growth (mm) of *P. oryzae* in the negative control plate, and *T* is the radial mycelia growth of *P. oryzae* in plates treated with *Trichoderma* spp.

On the other hand, pathogen viability was assessed by evaluating sporulation in a dual-plating experiment. The pathogen spores (10 days old) were collected by flooding the plates with 10 mL of sterile distilled water containing 0.05% Tween 80 and scraping with a rubber spatula. The spores were transferred to tubes containing 25 mL of distilled water, vortexed for 20 s to dislodge them, and then filtered through double-layer cheesecloth to remove mycelial fragments. The suspension was adjusted with distilled water to a final volume of 150 mL. A small aliquot (1 mL) was loaded into a hemocytometer under a light microscope for spore counting (spores/mL). The number of spores was recorded, and sporulation inhibition was calculated according to Equation 2 as described by Zhao et al. (2024).

$$\text{Sporulation Percenrage Inihibition}\;(\mathrm{PI})\%=\frac{S-T}{S}\times 100\;$$ (2)

Where *S* is the pathogen sporulation in control plates, and *T* is the pathogen sporulation in the presence of *Trichoderma* species.

#### 2.3.2 Activity 2: assessment of *P. oryzae* mycelial growth inhibition by non-volatile secondary metabolites

A bioassay for non-volatile compounds in *Trichoderma* was performed according to Singh and Singh (2019) method. *Trichoderma* species were cultured in an Erlenmeyer flask containing 100 mL of potato dextrose broth (PDB) and incubated in an orbital shaker at 100 rpm and 25 °C for 15 days. Then, the *Trichoderma* culture broth was filtered with a sterile Whatman filter No. 1 to remove the mycelia, and then sterilized through a sterile biological membrane filter (Hydrophilic PTFE biological filter, 0.45 μm and 0.22 μm). *Trichoderma* filtrate was mixed with PDA to obtain a concentration of 50% (v/v). A 7-day-old Pathogen mycelia plug (5 mm) was inoculated and incubated at 28 °C for 7 days. A positive control plate contained commercial *T. harzianum*, and a negative control plate contained PDA without *Trichoderma* filtrate. Treatments were assigned to a complete randomized design with four replications. Pathogen mycelial radial growth was measured every 24 h for up to 7 days, and the mycelial growth inhibition percentage was evaluated using Equation 1.

#### 2.3.3 Activity 3: assessment of *P. oryzae* mycelial growth inhibition by *Trichoderma* spp. in the dual culture bioassay

A dual culture bioassay, as described by Sriwati et al. (2019) and Steyaert et al., was done to determine the antagonistic capacity of *Trichoderma* spp. to inhibit *P. oryzae* mycelial growth. Petri dishes (90 mm) containing PDA were inoculated with a 5 mm disc of a 7-day-old pure culture of antagonistic fungi and pathogen in an opposite direction at a distance of 50 mm. Because of its low growth rate, *P. oryzae* inoculum was first incubated in darkness at 28 °C for 72 h; thereafter, *Trichoderma* spp. inoculum was planted at the opposite side, and the treatments were incubated at 28 °C for 7 days, making a total of 10 days of incubation time.

For the negative control, petri plates were inoculated with *P. oryzae* alone, and for the positive control, a commercial strain of *T. harzianum* was used. The choice of this *Trichoderma* spp. was based on reports that document its efficacy in managing novel fungal diseases and its wide adaptability (Yao et al., 2023). Four replications were assigned to each treatment in a completely randomized design (CRD). Radial mycelial growth of *P. oryzae* was measured daily for 7 days of co-incubation. Then, the percentage mycelial growth inhibition of *P. oryzae* was evaluated using Equation 1.

#### 2.3.4 Activity 4: assessment of *Trichoderma* mycoparasitism interaction by slide culture techniques

Mycoparasitic interactions were determined using a dual-slide culture technique as proposed by Saini et al. (2025).

to observe the types of interactions (coiling, penetration, or adhesion). Microcultures were prepared by sterilizing a 90-mm-diameter paper towel and placing it in a petri dish of the same diameter. Wooden sticks were arranged at the bottom, and sterilized slides were mounted on top. 150 μL of PDA was spread on the slides and allowed to solidify. A 7-day-old plug of *P. oryzae* was placed on one side of the slide culture opposite a 7-day-old culture of *Trichoderma* spp. The inoculum was covered and incubated at 28 °C for 5 days; then the interactions were observed under a light microscope at 20X and 40X magnification.

### 2.4 Evaluation of the degree of antagonism of *Trichoderma* spp.

The degree of antagonism of *Trichoderma* spp. against *P. oryzae* was assessed by using the antagonism scale 1–4 as proposed by Karličić et al. (2020). For the antagonism scale, 4- indicated very high antagonism (GI% > 75), 3- high antagonism (GI% 61–75), 2- moderate antagonism (GI% 51–60), and 1- low antagonism (GI% < 51). In addition, Bell’s competitive scale (Bell et al., 1982) was used to assess the competitive capacity of *Trichoderma* in a dual culture bioassay and was categorized into classes 1–5. Class 1—*Trichoderma* overgrew and covered the entire medium surface. Class 2—*Trichoderma* covered at least two-thirds of the medium surface. Class 3- Both *Trichoderma* and the pathogen colonized one-half of the medium surface. Class 4—The pathogen colonized at least two-thirds of the medium surface. Class 5—The pathogen completely overgrew *Trichoderma* and covered the entire medium surface. Lower mean scores ≤ 2 indicated strong antagonism, while higher mean scores ≥ 3 indicated weak antagonism.

## 3 Statistical analysis

Antagonistic data were analyzed using R software (version 4.3.2). Descriptive statistics (mean, standard deviation, standard error) were computed for each treatment, and inferential statistics were performed using one-way ANOVA. Significant differences among treatments were determined using Fisher’s LSD *post hoc* test at the 5% significance level. Percentage data were arcsine-transformed before analysis. A random forest classification model was additionally used to identify the most effective *Trichoderma* isolates.

## 4 Results

### 4.1 Identification of the *P. oryzae*

**Figure 1:**
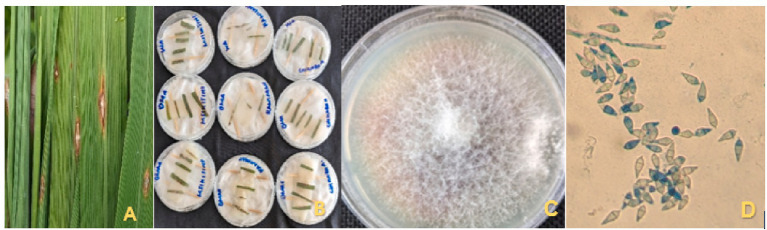
**(A)** Rice leaves with rice blast disease symptoms. **(B)** Isolation of *P. oryzae* on slide mount, **(C)** *P. oryzae* colony on PDA plate, **(D)** *P. oryzae* conidia on the light microscope.

*P. oryzae* was identified based on its cultural and microscopic characteristics. Colonies grown on oatmeal agar were initially white to light brown with a cottony texture. Microscopic examination revealed conidia pyriform (pear) with a distinct pedicel (stalk), three-celled conidia with two septa (Figure 1).

### 4.2 Inhibition of *P. oryzae* mycelial growth by *Trichoderma* spp. volatile organic compounds

**Figure 2:**
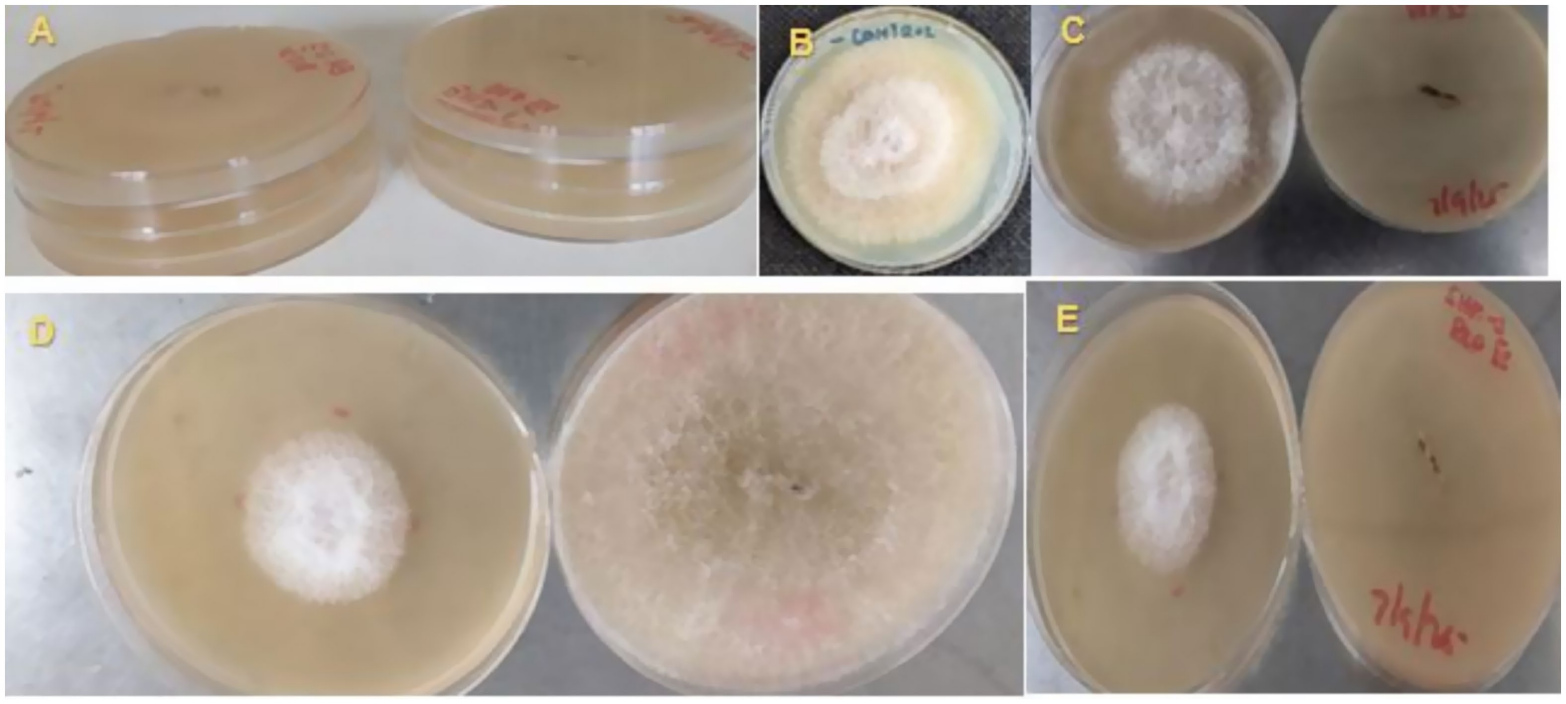
Growth inhibition of *P.oryzae* by VOCs: **(A)**. Dual plate technique, **(B)**. Growth of *P.oryzae* in the absence of antagonism, **(C–E)** growth of *P. oryzae* in the presence of antagonism.

**Figure 3:**
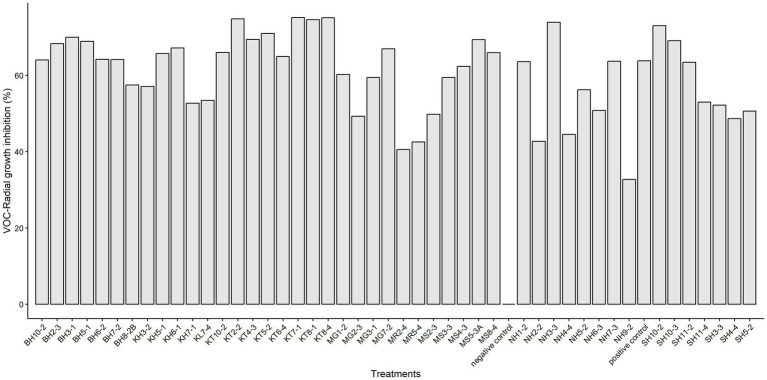
The effect of *Trichoderma* species volatile organic compounds on *P.oryzae* mycelial growth inhibition percentage.

Volatile compounds produced by *Trichoderma* species reduced the mycelial growth of *P. oryzae* (Figure 2). The growth inhibition percentage ranged from 42.70 to 75.11%, whereby the isolates *T. paraviridescens* KT7-1*, T. virens* KT8-4, *T. harzianum* (KT2-2, NH3-3, SH10-2, KT5-2), and *T. pleuroti* KT8-1 exhibited high growth rate inhibition of 70.96 to 75.11% (Figures 2, 3; Table 1). The lowest growth inhibition percentage was recorded for the isolates *T. virens* NH9-2 (32.70%), *T. harzianum* MR2-2 (40.57%), and *T. atroviride* MR5-4 (42.50%).

**Table 1:** *P. oryzae* mean values for radial growth and sporulation inhibition.

Treatments→ *Trichoderma* isolate↓	*P. oryzae* growth and sporulation inhibition			
	Dual culture_GI	Non-volatile compounds_GI	Volatile compounds (VOC)_GI	Volatile compounds (VOC)_PI
	Mean ± SED	Mean ± SED	Mean ± SED	Mean ± SED
BH10-2	1.01 ± 0.03 a	0.70 ± 0.02 ab	0.93 ± 0.07 abcd	0.71 ± 0.02 abcde
BH2-3	1.00 ± 0.03 a	0.69 ± 0.02 ab	0.98 ± 0.06 abcd	0.68 ± 0.03 abcde
BH3-1	1.04 ± 0.05 a	0.71 ± 0.03 ab	1.00 ± 0.05 abcd	0.74 ± 0.01 bcd
BH5-1	1.04 ± 0.02 a	0.72 ± 0.01 ab	0.98 ± 0.04 abcd	0.71 ± 0.01 abcde
BH6-2	0.93 ± 0.03 a	0.65 ± 0.02 ab	0.93 ± 0.04 abcd	0.73 ± 0.03 abcd
BH7-2	1.02 ± 0.02 a	0.58 ± 0.14 a	0.93 ± 0.03 abcd	0.70 ± 0.02 abcde
BH8-2B	0.99 ± 0.07 a	0.69 ± 0.04 ab	0.86 ± 0.04 abcde	0.67 ± 0.01 acde
KH3-2	1.02 ± 0.03 a	0.71 ± 0.01 ab	0.86 ± 0.03 abcde	0.67 ± 0.01 acde
KH5-1	0.95 ± 0.04 a	0.67 ± 0.01 ab	0.95 ± 0.08 abcd	0.69 ± 0.01 abcde
KH6-1	1.02 ± 0.05 a	0.69 ± 0.03 ab	0.97 ± 0.08 abcd	0.73 ± 0.03 bcd
KH7-1	1.08 ± 0.03 a	0.73 ± 0.02 ab	0.81 ± 0.06 abcde	0.74 ± 0.03 bcd
KL7-4	1.00 ± 0.04 a	0.69 ± 0.02 ab	0.82 ± 0.05 abcde	0.59 ± 0.02 ae
KT10-2	1.05 ± 0.03 a	0.71 ± 0.01 ab	0.95 ± 0.04 abcd	0.70 ± 0.02 abcde
KT2-2	1.03 ± 0.19 a	0.71 ± 0.01 ab	1.05 ± 0.05 a	0.59 ± 0.07 e
KT4-3	1.00 ± 0.04 a	0.69 ± 0.02 ab	0.99 ± 0.06 abcd	0.68 ± 0.05 abcde
KT5-2	1.04 ± 0.02 a	0.72 ± 0.01 ab	1.01 ± 0.06 abc	0.75 ± 0.02 bcd
KT6-4	0.97 ± 0.04 a	0.66 ± 0.02 ab	0.94 ± 0.04 abcd	0.59 ± 0.02 ae
KT7-1	1.02 ± 0.04 a	0.70 ± 0.02 ab	1.07 ± 0.09 a	0.81 ± 0.01 b
KT8-1	1.03 ± 0.03 a	0.71 ± 0.01 ab	1.05 ± 0.06 a	0.79 ± 0.00 bc
KT8-4	1.10 ± 0.04 a	0.74 ± 0.02 b	1.06 ± 0.05 a	0.76 ± 0.02 bc
MG1-2	1.07 ± 0.01 a	0.72 ± 0.01 ab	0.89 ± 0.07 abcde	0.70 ± 0.04 abcde
MG2-3	0.97 ± 0.04 a	0.68 ± 0.02 ab	0.78 ± 0.04 abcde	0.69 ± 0.02 abcde
MG3-1	1.06 ± 0.02 a	0.72 ± 0.01 ab	0.88 ± 0.07 abcde	0.79 ± 0.01 bc
MG7-2	1.02 ± 0.02 a	0.70 ± 0.01 ab	0.96 ± 0.06 abcd	0.66 ± 0.01 acde
MR2-4	1.07 ± 0.06 a	0.72 ± 0.03 ab	0.69 ± 0.01 de	0.67 ± 0.00 acde
MR5-4	1.06 ± 0.03 a	0.72 ± 0.01 ab	0.71 ± 0.05 cde	0.62 ± 0.01 ade
MS2-3	1.04 ± 0.03 a	0.72 ± 0.01 ab	0.78 ± 0.04 abcde	0.71 ± 0.02 abcde
MS3-3	1.00 ± 0.05 a	0.70 ± 0.02 ab	0.88 ± 0.03 abcde	0.70 ± 0.02 abcde
MS4-3	1.07 ± 0.05 a	0.73 ± 0.02 ab	0.91 ± 0.02 abcde	0.74 ± 0.01 bcd
MS5-3A	0.98 ± 0.06 a	0.67 ± 0.03 ab	0.99 ± 0.04 abcd	0.67 ± 0.01 acde
MS8-4	1.06 ± 0.04 a	0.72 ± 0.02 ab	0.95 ± 0.05 abcd	0.69 ± 0.02 abcde
NH1-2	1.07 ± 0.01 a	0.73 ± 0.01 ab	0.92 ± 0.03 abcd	0.72 ± 0.02 abcde
NH2-2	1.07 ± 0.04 a	0.73 ± 0.01 ab	0.71 ± 0.04 cde	0.69 ± 0.01 abcde
NH3-3	1.12 ± 0.04 a	0.75 ± 0.02 b	1.04 ± 0.04 a	0.74 ± 0.01 bcd
NH4-4	1.03 ± 0.02 a	0.71 ± 0.01 ab	0.73 ± 0.02 bcde	0.72 ± 0.01 abcde
NH5-2	1.07 ± 0.04 a	0.73 ± 0.01 ab	0.86 ± 0.08 abcde	0.68 ± 0.01 abcde
NH6-3	1.06 ± 0.01 a	0.72 ± 0.01 ab	0.80 ± 0.09 abcde	0.72 ± 0.02 abcde
NH7-3	1.15 ± 0.04 a	0.77 ± 0.02 b	0.93 ± 0.04 abcd	0.72 ± 0.01 abcde
NH9-2	1.13 ± 0.04 a	0.75 ± 0.01 b	0.61 ± 0.05 e	0.69 ± 0.01 abcde
SH10-2	1.01 ± 0.08 a	0.69 ± 0.04 ab	1.04 ± 0.07 ab	0.78 ± 0.01 bc
SH10-3	1.00 ± 0.06 a	0.69 ± 0.03 ab	0.99 ± 0.07 abcd	0.81 ± 0.05 b
SH11-2	1.00 ± 0.03 a	0.68 ± 0.01 ab	0.93 ± 0.07 abcd	0.77 ± 0.03 bc
SH11-4	1.13 ± 0.04 a	0.75 ± 0.01 b	0.81 ± 0.03 abcde	0.76 ± 0.03 bc
SH3-3	1.05 ± 0.04 a	0.71 ± 0.02 ab	0.81 ± 0.03 abcde	0.68 ± 0.02 abcde
SH4-4	1.01 ± 0.05 a	0.69 ± 0.02 ab	0.77 ± 0.09 abcde	0.71 ± 0.03 abcde
SH5-2	1.00 ± 0.07 a	0.70 ± 0.04 ab	0.79 ± 0.03 abcde	0.67 ± 0.02 acde
Negative control	0.00 ± 0.00 b	0.00 ± 0.00 c	0.00 ± 0.00 f	0.00 ± 0.00 f
Positive control	0.94 ± 0.04 a	0.67 ± 0.01 ab	0.93 ± 0.04 abcd	0.73 ± 0.04 abcd
	SED = 0.056; LSD = 0.110	SED = 0.040; LSD = 0.078.	SED = 0.075; LSD 0.149	SED = 0.033; LSD = 0.066

Letters (a–f) within the same column indicate significant differences among treatments. The means followed by the same letter are not significantly different at *p* ≤ 0.05 according to Fisher’s LSD test.

Treatment means were significant (ANOVA: *F* = 47.14; *p* < 0.001), and they ranged from 0.61 ± 0.05 to 1.07 ± 0.09 (mean ± SE) (Table 1). The highest responses were recorded from *T. paraviridescens KT*7-1, *T. virens* KT8-4, *T. pleuroti* KT8-1 *and T. harzianum* (KT2-2, SH10-2, and NH3-3) with of 1.07 ± 0.09 to 1.04 ± 0.04 (Table 1). These species performed better than commercialized *T. harzianum* (0.93 ± 0.04). The lowest responses were recorded for the treatments *T. virens* NH9-2(0.61 ± 0.05) and *T. harzianum,* MR2-4(0.69 ± 0.01). The rest of the treatments showed intermediate performance, with means between 0.71 ± 0.05 and 1.01 ± 0.06.

**Figure 4:**
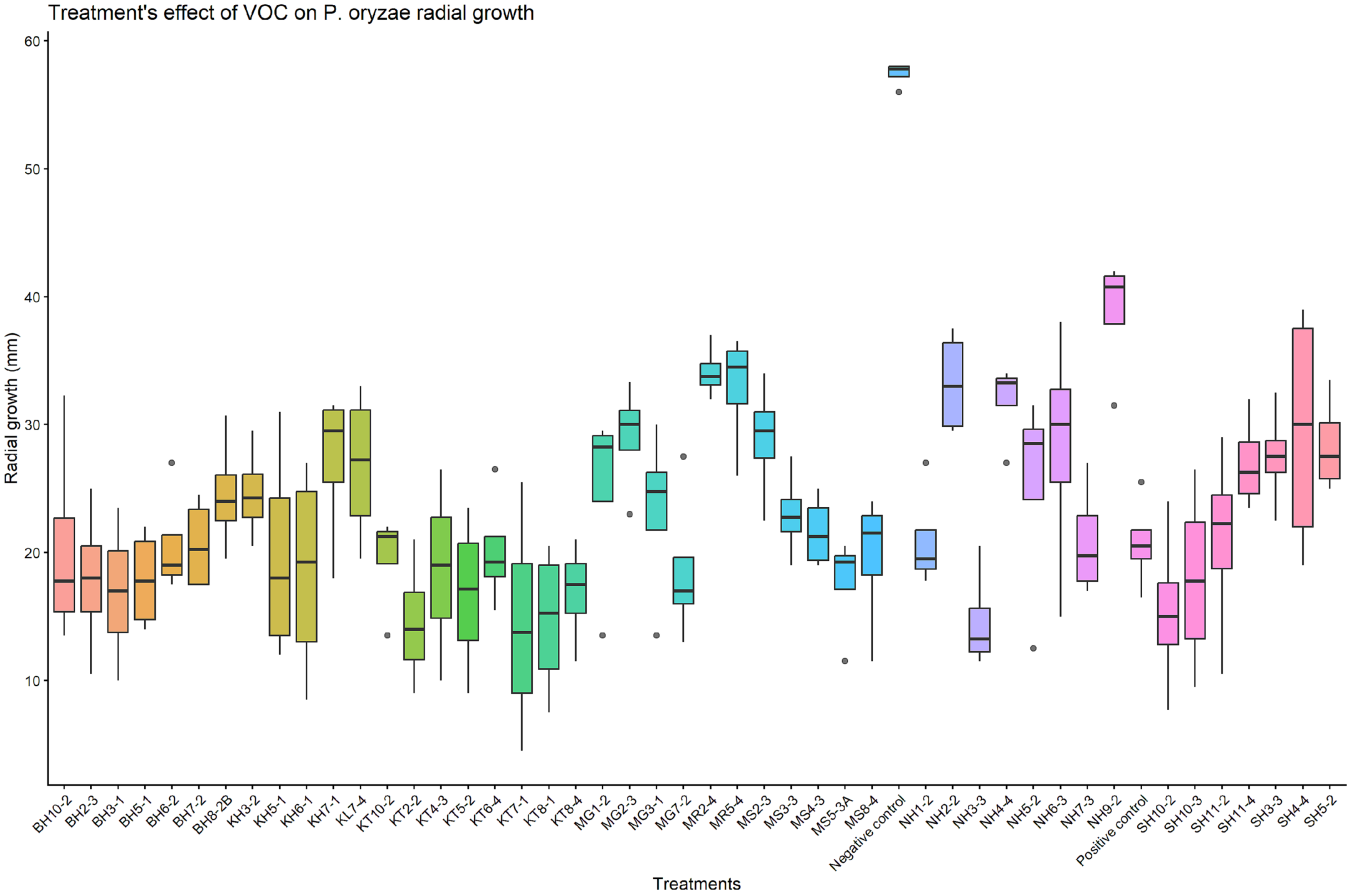
Effects of *Trichoderma* spp. volatile organic compounds on *P. oryzae* mycelial radial growth.

Analysis of colony radial growth further explained the antagonistic effects of *Trichoderma* VOCs on *P. oryzae* (Figure 4). The negative control (*P. oryzae* only) had the highest colony radial growth compared to the *Trichoderma* treatments. The lowest *P. oryzae* radial growth was observed for *Trichoderma* isolate *T. harzianum* SH10-2, KT2-2, and NH3-3, KT7-1(*T. paraviridescens*), *and KT8-1*(*T. pleuroti*), while *T. virens* NH9-2, *T. harzianum* MR2-4, and NH4-4 exhibited relatively higher *P. oryzae* radial growth, indicating weak antagonistic effects of VOC from these isolates. The commercial *T. harzianum* expressed an intermediate level of inhibition, as did most *Trichoderma* isolates.

### 4.3 Inhibition of *P. oryzae* sporulation by *Trichoderma* volatile organic compounds

**Figure 5:**
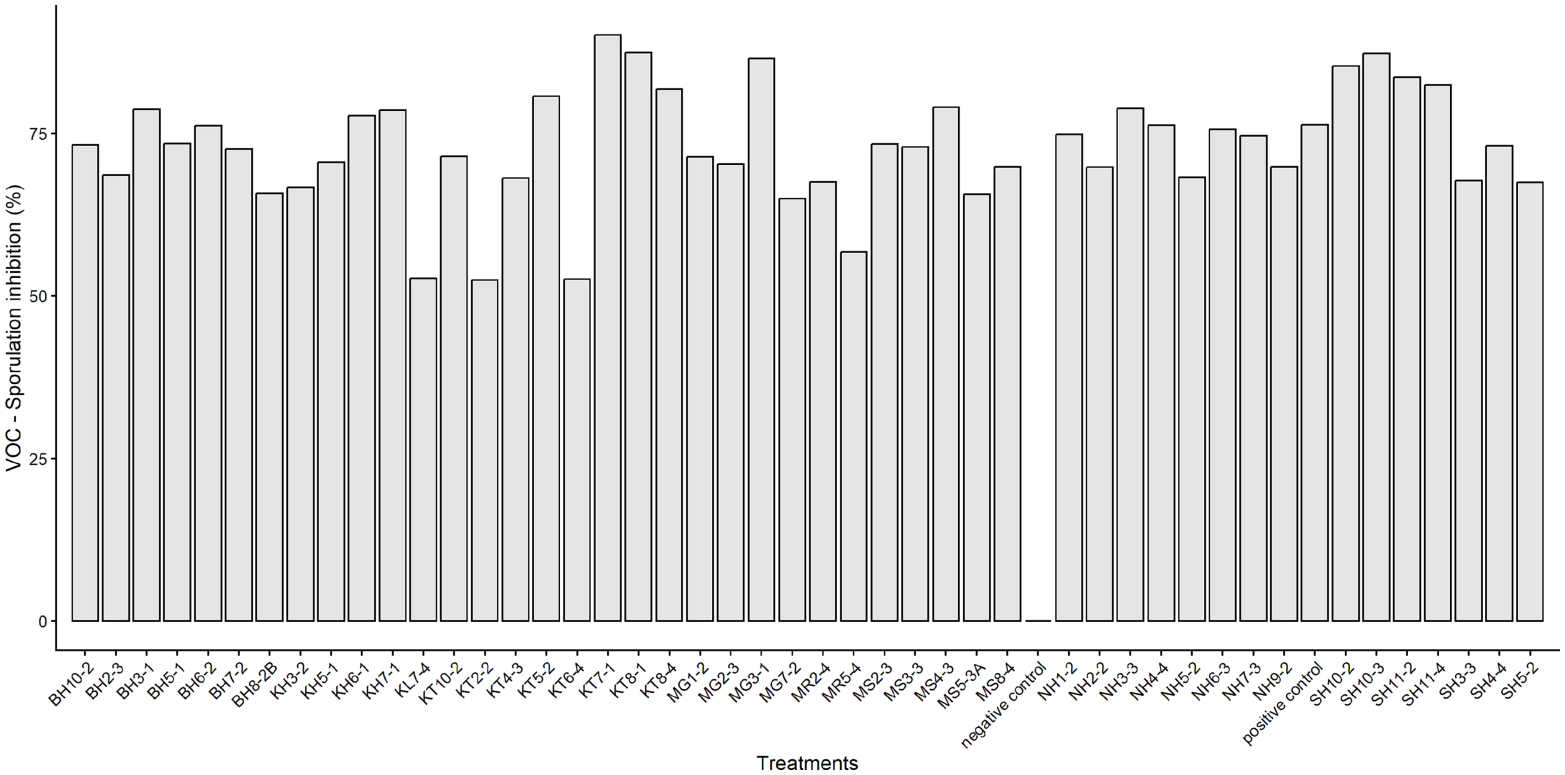
The effect of *Trichoderma* species volatile organic compounds on *P.oryzae* spore inhibition percentage.

The results of the study demonstrated significant variation in the inhibition of *P. oryzae* sporulation by volatile organic compounds (VOCs) emitted by different *Trichoderma* isolates (Figure 5). Inhibition rates ranged from 52.47 to 90.12%. The isolates of *T. paraviridescens* KT7-1, *T. pleuroti* KT8-1, *T. harzianum* (SH10-3, SH10-2, SH11-2, KT5-2), *T. erinaceum* MG3-1, and *T.virens* KT8-4 exhibited the highest inhibitory activity (82.68–90.12%), surpassing the positive control, a commercial *T. harzianum* strain (76.36%). In contrast, the lowest inhibition was observed for *T. harzianum* KT2-2, KT6-4, and KL7-4 with 52.47, 52.59, and 52.72%, respectively, and *T. atroviride* MR5-4 (56.76%).

The analysis confirmed significant differences in sporulation inhibition among all treatments (*F* = 47, 144; *p* < 0.001). Pairwise comparisons using Fisher’s LSD test revealed distinct efficacy groups. The most effective isolates (SH10-3, MG3-1, SH10-2, SH11-2, SHH11-4, and KT5-2) formed a statistically superior group, corresponding to mean inhibition values of 0.81 ± 0.01 to 0.75 ± 0.02 (Table 1). Conversely, the least effective isolates (KT6-4, KL7-4, and KT2-2) formed a separate group with significantly lower mean inhibition values of 0.59 ± 0.02 to 0.59 ± 0.07. All treatments resulted in significantly higher inhibition compared to the negative control (0.00 ± 0.00).

**Figure 6:**
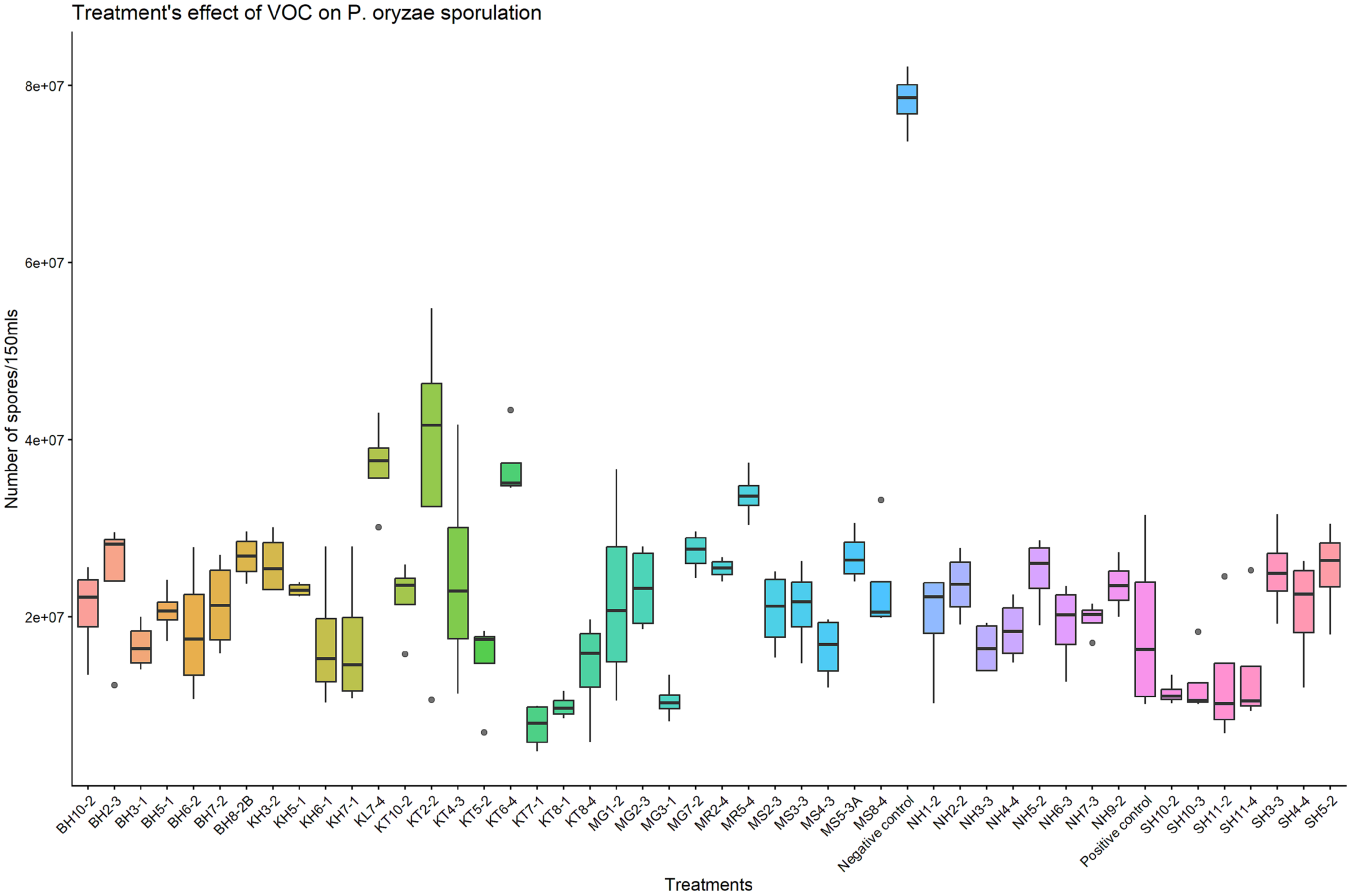
Effects of *Trichoderma* spp. volatile organic compounds on *P. oryzae* spore production.

Analysis of spore production by *P. oryzae* further quantified the inhibitory effects of *Trichoderma* VOCs (Figure 6). The negative control yielded the highest spore count at 7.8 × 10^7^ spores per 150 mL. Among the treatments, isolates KT2-2, KT6-4, and KL7-4 exhibited the weakest sporulation inhibition, with spore production at 3.7 × 10^7^ spores per 150 mL each. The most effective inhibition was observed for isolates SH10-3, SH10-2, SH11-4, and MG3-1, which reduced spore production to (1.1 × 10^7^, 1.2 × 10^7^, 1.3 × 10^7^, and 1.4 × 10^7^) spore/ 150 mL and significantly suppressed sporulation. The efficacy of these top-performing isolates exceeded that of the commercial *T. harzianum* positive control, which resulted in 1.8 × 10^7^ spores per 150 mL.

### 4.4 Antagonistic potential of *Trichoderma* non-volatile compounds on *P. oryzae* radial growth

**Figure 7:**
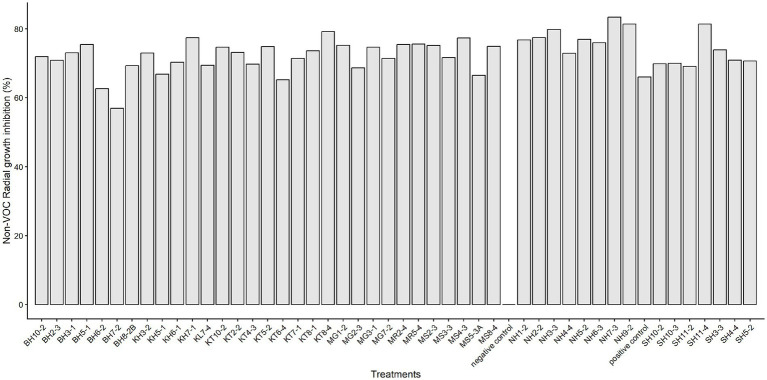
The effect of *Trichoderma* species non-volatile organic compounds on *P.oryzae* mycelial growth inhibition percentage.

The production of diffusible (non-volatile) organic compounds by *Trichoderma* species resulted in significant inhibition of *P. oryzae* colony radial growth, with efficacy ranging from 56.89 to 83.35% (Figure 7). The strongest antagonistic activity was exhibited by isolates *T. virens* (NH7-3, KT8-4, and NH9-2), and *T. harzianum* (SH11-4, NH3-3) with inhibition values of 79.21 to 83.35%. The inhibition by these top performers exceeded that of the commercial *T. harzianum* positive control (65.98%). The lowest inhibition was recorded for *T. harzianum* BH7-2 (56.89%).

The analysis confirmed significant differences in radial growth inhibition among all treatments (*F* = 14.37; *p* < 0.001). Pairwise comparisons using Fisher’s LSD test revealed distinct efficacy groups. The most effective isolates (NH7-3, NH9-2, SH11-4, and NH3-3) formed a statistically superior group, corresponding to mean inhibition values of 0.77 ± 0.02 to 0.75 ± 0.02 (Table 1). On the contrary, the least effective isolate, *T. harzianum* BH7-2, formed a separate group with significantly lower mean inhibition values of 0.58 ± 0.14. However, all treatments resulted in significantly higher inhibition compared to the negative control (0.00 ± 0.00).

**Figure 8:**
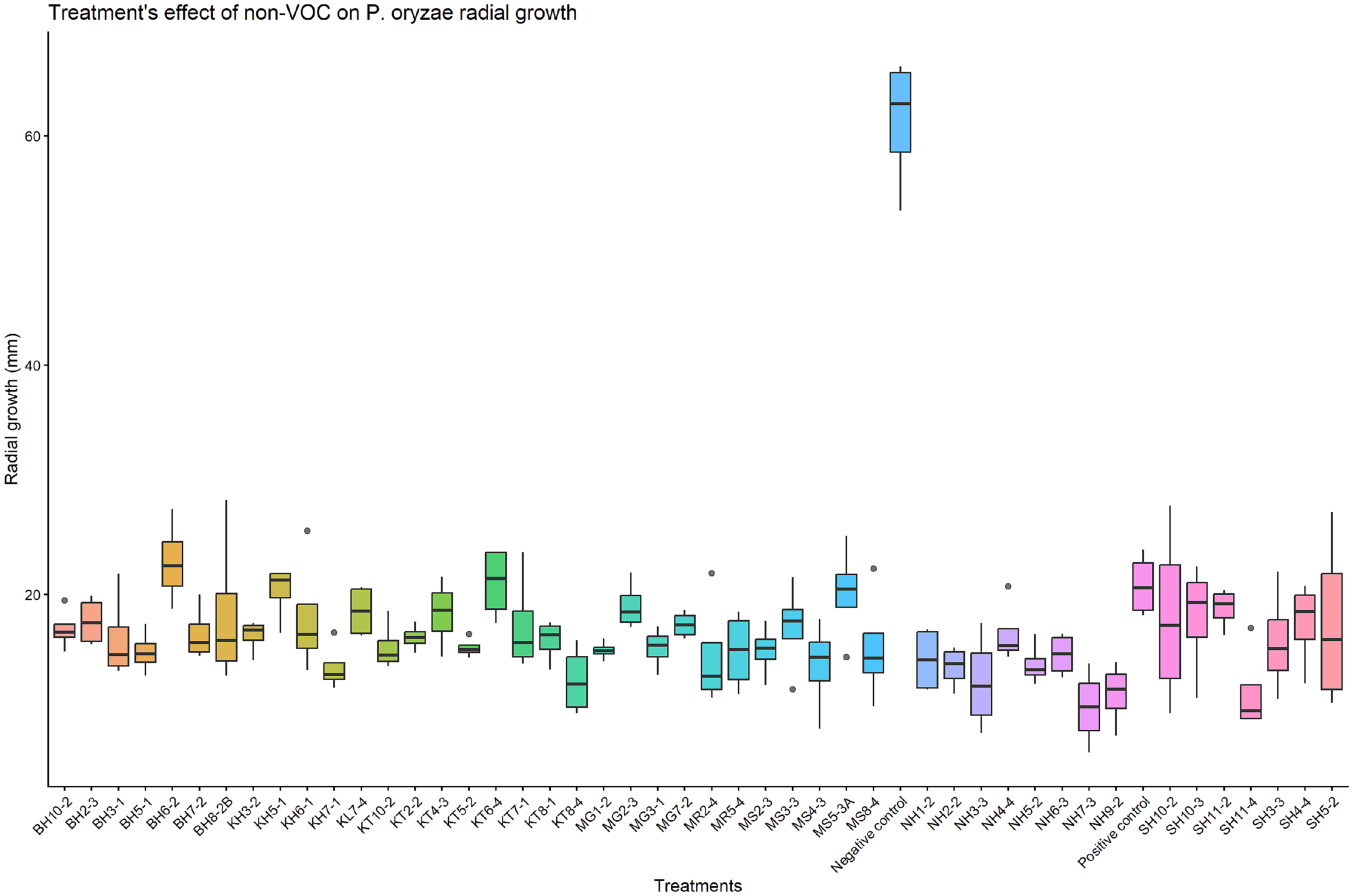
Effects of *Trichoderma* spp. non-volatile organic compounds on *P. oryzae* mycelial radial growth.

Analysis of colony radial growth further explained the antagonistic effects of *Trichoderma* non-VOCs on *P. oryzae* (Figure 8). The negative control (*P. oryzae* only) had the highest colony radial growth compared to the *Trichoderma* treatments. The lowest *P. oryzae* radial growth was observed for *Trichoderma* isolate SH11-4, NH9-2, NH7-3, and NH3-3, while isolates BH6-2, KH5-1, KT6-4, and MS5-3A exhibited relatively higher *P. oryzae* radial growth, indicating weak antagonistic effects of non-VOCs from these isolates. The commercial *T. harzianum* expressed an intermediate level of inhibition, as did most *Trichoderma* isolates.

### 4.5 Antagonistic potential of *Trichoderma* species in dual culture bioassay

**Figure 9:**
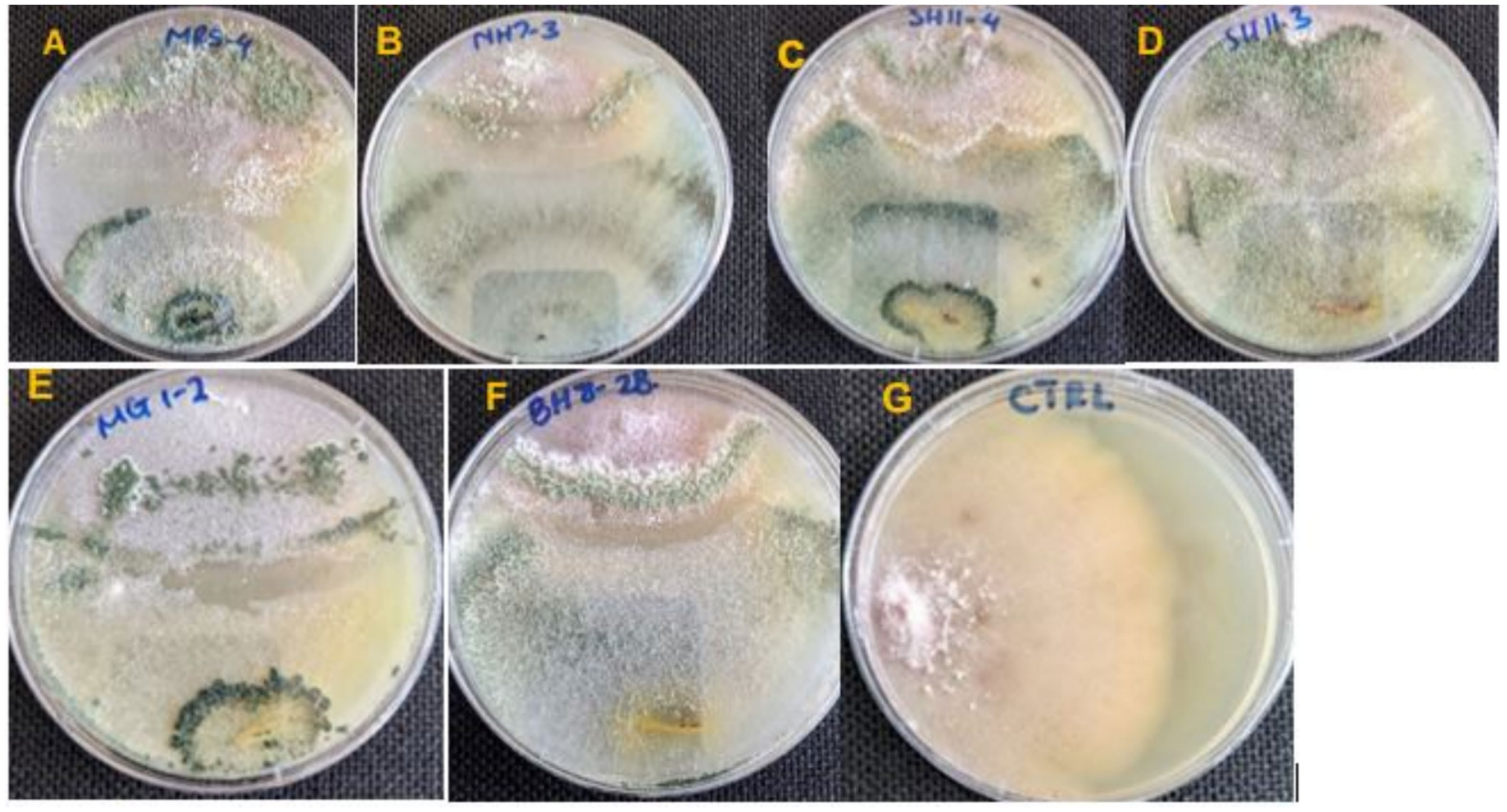
Antagonistic activity of *Trichoderma* species in dual culture; **(A)**. *T. atroviride*, **(B)**. *T. virens*, **(C–E)**, and *T. harzianum,* and **(G)** is the *P. oryzae* alone.

**Figure 10:**
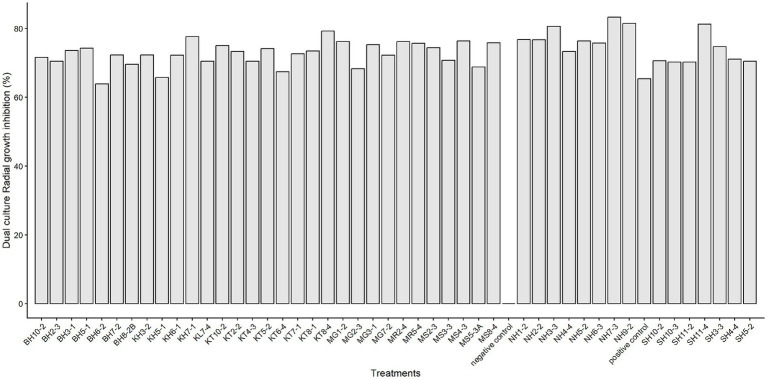
The effect of *Trichoderma* species on *P. oryzae mycelial* growth inhibition percentage through direct antagonism.

*Trichoderma* isolates exhibited varying levels of efficiency in suppressing *P. oryzae* mycelial growth from day three to seven of co-culture (Figures 9, 10), with growth inhibition percentages ranging from 63.87 to 83.25%. The highest antagonistic activity was recorded for *T. virens* (NH7-3, NH9-2) and *T. harzianum* (SH11-4, NH3-3), with inhibition levels of 80.58 to 83.25%, and was higher compared to the commercial *T. harzianum* positive control (65.37%). In contrast, *T. harzianum* BH6-2 showed the lowest inhibition of 63.87%.

Statistical analysis revealed a highly significant treatment effect on growth inhibition in dual culture (*F* = 47, *p* < 0.001). Mean growth inhibition values ranged from 0.93 ± 0.03 to 1.15 ± 0.04, compared with the negative control (0.00 ± 0.00). Although all isolates belonged to the same statistical group, the isolates NH7-3, NH9-2, SH11-4, and NH3-3 (1.12 ± 0.04 to 1.15 ± 0.04) performed better compared to other isolates. BH6-2, and the commercial *T*. *harzianum* showed lower mean values (0.93 ± 0.03 and 0.94 ± 0.04, respectively).

**Figure 11:**
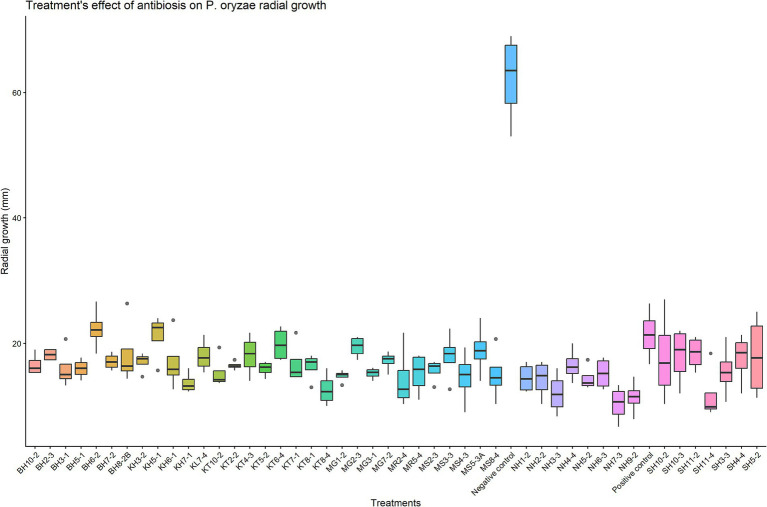
Effects of *Trichoderma* direct antagonism on *P. oryzae* colony radial growth.

Boxplot analysis of *P. oryzae* radial growth (Figure 11) revealed a variation among *Trichoderma* treatments. The negative control showed the highest radial growth, indicating uninhibited pathogen development. In contrast, *T. harzianum* (SH11-4 and NH3-3) and *T. virens* (NH7-3 and NH9-2) consistently resulted in the lowest radial growth, reflecting strong antagonistic activity. Moderate pathogen growth was observed with KT8-4 (*T. virens*) and MR2-4 (*T. harzianum*), whereas BH6-2, KH5-1 (*T. harzianum*), and the commercial *T. harzianum* positive control exhibited relatively higher radial growth.

#### 4.5.1 *Trichoderma* spp.–*P. oryzae* mycoparasitism relationship

**Figure 12:**
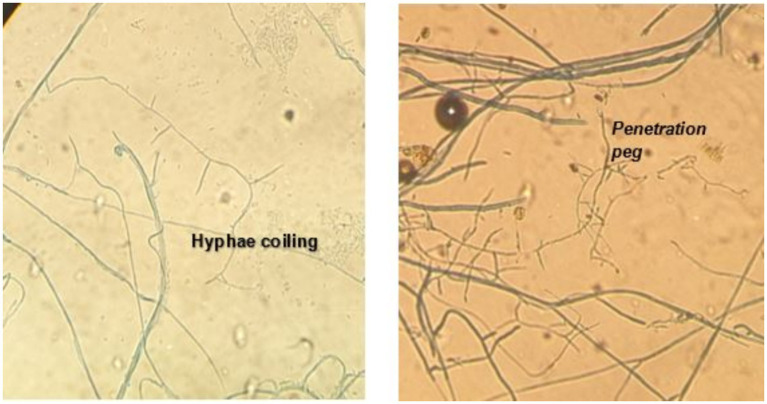
Mycoparasitism of *Trichoderma* species against *P. oryzae* hyphae.

*Trichoderma*–*P. oryzae* mycoparasitic interaction was evident 3 days after co-culture (Figure 12). *Trichoderma* isolates exhibited clear mycoparasitic behavior, characterized by coiling of their hyphae around *P. oryzae* hyphae, formation of penetration pegs, and adhesion to the pathogen’s hyphal surface, indicating direct parasitism of the phytopathogen. However, no visible mycoparasitic interaction (coiling, penetration, or adhesion) was observed for the *T. virens* isolate under the same conditions.

#### 4.5.2 Evaluation of the degree of antagonism of *Trichoderma* spp.

**Figure 13:**
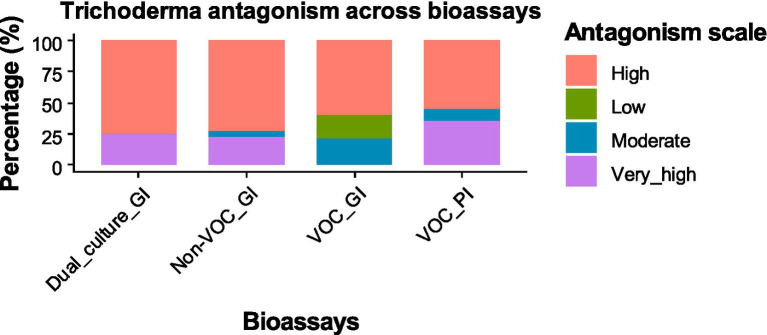
*Trichoderma* species antagonism activities across the bioassays.

**Figure 14:**
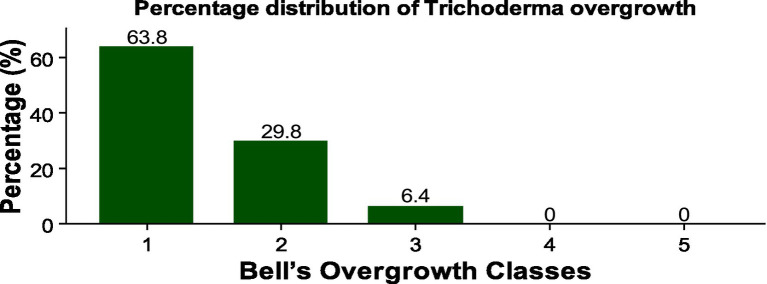
*Trichoderma* species competitive classes.

All evaluated *Trichoderma* isolates demonstrated strong antagonistic activity against *P. oryzae* in all bioassays. However, the degree of antagonism varied among species and among bioassays (Figure 13). In the dual culture and non-volatile assays, most isolates exhibited high growth inhibition (GI = 61–75%) or very high growth inhibition (GI > 75%). Volatile organic compound (VOCs) assays showed a wider variation in both mycelial growth and sporulation inhibition. For growth inhibition, VOCs from *Trichoderma* isolates were categorized as low inhibitors (GI < 51%), moderate inhibitors (GI = 51–60%), and high inhibitors (GI = 61–75%). For sporulation inhibition, VOCs acted as moderate inhibitors (PI = 51–60%), high inhibitors (PI = 61–75%), and very high inhibitors (PI > 75%), indicating that VOCs were more effective in inhibiting spore production than in reducing mycelial radial growth. In dual culture, isolates exhibited strong competitiveness for space and nutrients, whereby 93.6% of *Trichoderma* species had a mean score value of ≤2, indicating being strong competitors (Figure 14).

**Figure 15:**
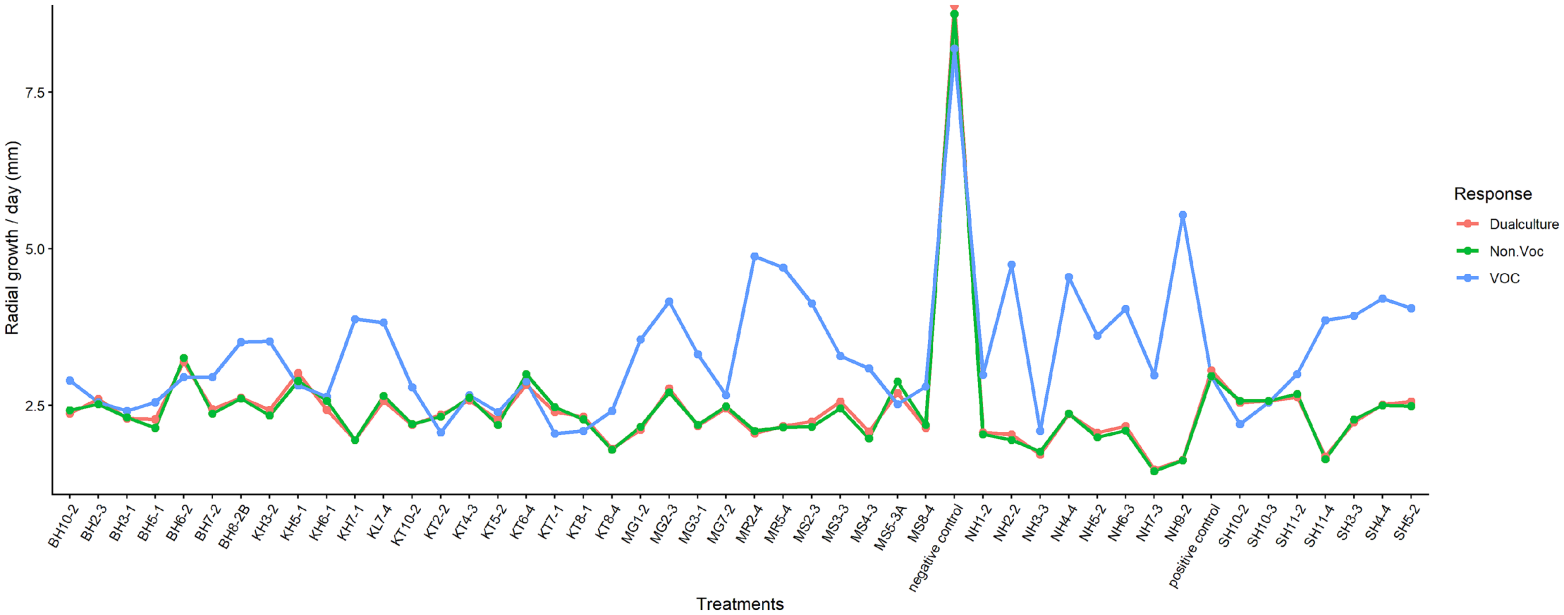
Comparable performance of antagonism mechanisms in inhibiting *P. oryzae* growth.

On the other hand, the comparison of *P. oryzae* radial growth rate (mm/day) across the three antagonism mechanisms [dual culture, non-volatile organic compounds (Non-VOCs)], and volatile organic compounds (VOCs) was evaluated (Figure 15). The dual-culture and non-volatile metabolite (Non-VOC) bioassays showed a consistently low *P. oryzae* growth rate that typically ranged from 1.7 to 3.0 mm day^−1^ indicating strong suppression through direct contact and diffusible metabolites. VOC-mediated inhibition was low compared to the non-VOC and dual culture bioassay and was highly variable within *Trichoderma* species. The negative control exhibited the highest growth (8 mm day^−1^), confirming the natural aggressiveness of *P. oryzae* in the absence of antagonism. Although its performance was lower compared to indigenous *Trichoderma* species, the commercial *T. harzianum*, serving as the positive control, consistently inhibited pathogen growth across all bioassays.

**Figure 16:**
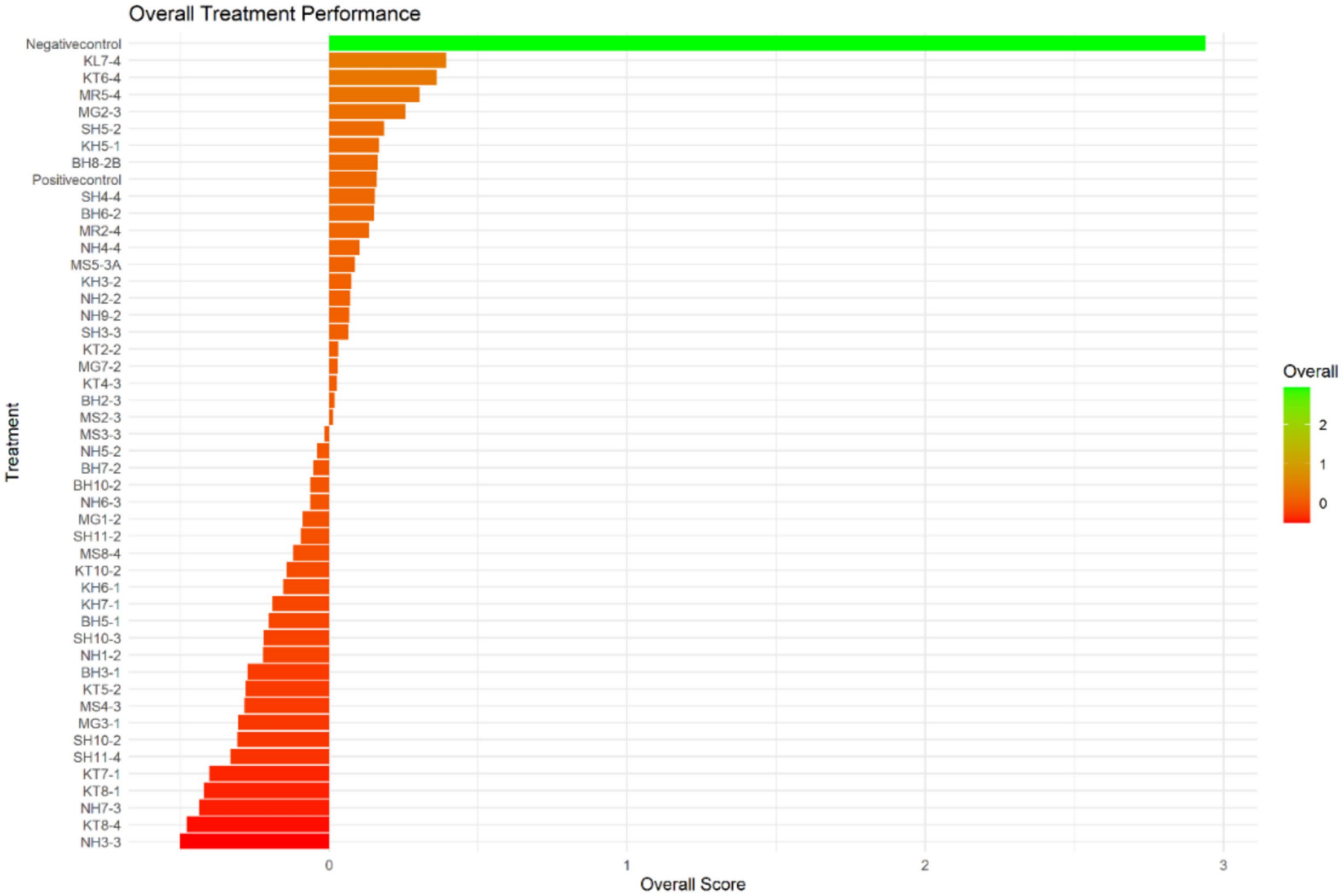
Overall performance of *Trichoderma* species across the three antagonism experiments.

The random forest classification model ranked the overall performance of all *Trichoderma* species against *P. oryzae* across the different bioassays (Figure 16). Positioned at the lower end of the plot (bottom left) correspond to the best overall performers, showing consistently high inhibition of pathogen growth and/or sporulation across all bioassays. In contrast, isolates located toward the upper part of the plot represent comparatively lower performers, with lower predicted antagonistic efficiency and reduced contribution to overall biocontrol potential.

## 5 Discussion

This study provides a comprehensive evaluation of the antagonistic potential of diverse *Trichoderma* isolates against the rice blast pathogen *P. oryzae* by integrating direct interactions, diffusible metabolites, and volatile-mediated mechanisms. While all tested isolates suppressed pathogen growth and sporulation *in vitro*, the magnitude and consistency of inhibition varied significantly by species, strains, and mode of antagonism, demonstrating that *Trichoderma* antagonism was highly isolate- and mechanism-dependent, reflecting the complex and specialized nature of *Trichoderma*–pathogen interactions.

### 5.1 Volatile-mediated antagonism

The VOCs bioassays showed that *Trichoderma* spp. can suppress *P. oryzae* growth and sporulation without physical contact, confirming the importance of volatile-mediated antibiosis. However, substantial interspecific and intraspecific variability was observed. Isolates such as *T. harzianum* SH10-3, SH10-2, SH11-2, SH11-4, KT5-2; *T. virens* KT8-4; *T. pleuroti* KT8-1; *T. erinaceum* MG3-1; and *T. paraviridescens* KT7-1 exhibited strong VOC-mediated inhibition, whereas the commercial *T. harzianum* strain and isolates MR2-4, KL7-4, NH4-4, KT2-2, KT6-4, and *T. virens* NH9-2 were less effective. These differences suggest variation in the quantity, composition, or bioactivity of emitted VOCs.

These inhibitory effects are attributable to a broad spectrum of volatile compounds, including alcohols, phenols, ketones, lactones/pyrones, hydrocarbons, terpenes, aldehydes, and thioesters. Volatile compounds produced by biocontrol microorganisms such as *Trichoderma* spp. contribute to pathogen inhibition by diffusing through air or soil pores and interfering with pathogens’ essential cellular processes. These processes include fungal respiration, membrane integrity, mycelial growth, and spore germination. Previous studies have similarly reported strong VOCs production in *T. harzianum, T. virens, T. longibrachiatum, and T. asperellum* (Joo and Hussein, 2022; Li et al., 2024). Notably, 6-pentyl-2H-pyran-2-one (6-PP), produced by *T. atroviride*, has been shown to suppress *Fusarium wilt* while promoting plant growth (Rao et al., 2022), and VOCs such as 2-pentylfuran, acetophenone, and p-cymene, produced by *Trichoderma* spp., have demonstrated both antimicrobial activity and induction of plant resistance (Gualtieri et al., 2022).

### 5.2 Physical and non-volatile mediated antagonism mechanisms

The present study demonstrates that *Trichoderma* spp. suppressed *P. oryzae* through a coordinated interplay of physical interactions and chemical antagonism, as evidenced by both dual culture confrontation and culture filtrate (non-volatile metabolite) bioassays. Across all isolates, strong inhibition of pathogen growth was observed, confirming the broad biocontrol potential of the genus. Notably, isolate and species-dependent variation highlights the mechanistic diversity underlying antagonistic performance.

In dual-culture assays, all *Trichoderma* isolates exhibited strong antagonistic activity *against P. oryzae*, confirming a generally high inhibitory potential across the genus. However, quantitative differences were significant. *T. virens* NH7-3 and NH9-2, together with *T. harzianum* SH11-4 and NH3-3, showed the highest inhibition (80.58–83.25%), followed by several moderately effective isolates (76.16–79.25%). The commercial *T. harzianum* strain and isolate *T. harzianum* BH6-2 exhibited the lowest inhibition values. The high levels of inhibition observed in dual culture are attributed to the synergistic integration of mycoparasitism, antibiosis, and aggressive competition for space and nutrients (Kumari et al., 2025; Sarria et al., 2021). *Trichoderma* spp. are widely recognized for their rapid colonization capacity (Manzar et al., 2022), which effectively complements both contact-dependent and chemically mediated antagonistic mechanisms (Sood et al., 2020). Owing to their fast growth rate, extensive mycelial development, and prolific sporulation (Kumari et al., 2025; Sarria et al., 2021; Sood et al., 2020).

Comparable antagonistic efficacy of *Trichoderma* spp. against *P. oryzae* has been widely reported. Prismantoro et al. (2024) observed that *T. yunnanense* TM10 inhibited *P. oryzae* by 86.08%. Similarly, strong bioefficacy of *T. viride, T. virens, T. harzianum*, and *T. sulphureum* against major rice pathogens, including *P. oryzae* (rice blast), *Sarocladium oryzae* (sheath rot), and *Bipolaris oryzae* (brown spot), has been documented (Kulmitra et al., 2017; Lukanda et al., 2024).

Microscopic observations confirmed direct mycoparasitic interactions, including hyphal contact, coiling around *P. oryzae* hyphae, and the formation of penetration pegs. These structures are characteristic of *Trichoderma-*mediated mycoparasitism, indicating active recognition and attack of the pathogen (Sarria et al., 2021). Upon physical contact, *Trichoderma* spp. secrete cell wall–degrading enzymes (CWDEs) such as chitinases, β-1,3-glucanases, proteases, and cellulases, which hydrolyze key structural polymers of the pathogen cell wall, weaken hyphal integrity, and ultimately cause cell collapse and death (Mukherjee et al., 2025). Isolates exhibiting stronger antagonism in dual culture likely possess enhanced enzymatic activity, particularly in the production of cell wall–degrading enzymes (CWDEs) such as chitinases, β-1,3-glucanases, and proteases (Ghasemi et al., 2020; Kappel et al., 2024). Consistent with previous reports, elevated CWDEs production has been closely associated with superior pathogen suppression, explaining the variability in antagonistic performance observed among *Trichoderma* spp. (Garcés-Moncayo et al., 2025b).

In addition to contact-dependent effects, chemical antagonism mediated by non-volatile secondary metabolites played a crucial role in inhibiting *P. oryzae*, as demonstrated by the culture filtrate bioassays. Non-volatile metabolites reduced and inhibited pathogen growth by 56.68–83.35%. Among the tested isolates, *T. virens* NH7-3, NH9-2, and *T. harzianum* SH11-4, NH3-3 consistently exhibited the strongest antagonistic activity (79.21–83.35%). The strong suppression observed in the absence of physical contact confirms that diffusible metabolites are efficient enough to significantly restrict pathogen growth, reinforcing their importance in *Trichoderma*-based biocontrol.

*Trichoderma* spp. are known to produce a wide array of antifungal non-volatile compounds, including peptaibols, polyketides, terpenoids, and related antibiotics (Birari et al., 2025), which interfere with membrane integrity, protein synthesis, respiration, and spore germination, thereby reinforcing growth inhibition at the interaction zone (Garcés-Moncayo et al., 2025a; Rao et al., 2022). Non-volatile antibiotics such as gliotoxin, viridin, and peptaibols further enhance antagonism by disrupting mitochondrial function and cellular metabolism, leading to growth arrest and pathogen death (Birari et al., 2025; Li et al., 2019).

Among the most important non-volatile metabolites are peptaibols, membrane-active peptide antibiotics that disrupt pathogen cells by inserting into lipid bilayers via their amphipathic *α*-helical structure. Their hydrophobic regions interact with membrane lipids while the hydrophilic regions facilitate channel formation, allowing peptaibols to aggregate into transmembrane pores. These pores increase membrane permeability, causing ion and metabolite leakage, collapse of proton gradients, and disruption of cellular energetics, ultimately leading to loss of membrane potential, cellular homeostasis, and pathogen cell lysis (Birari et al., 2025; Hou et al., 2022).

*Trichoderma virens* and *T. harzianum* are well-known producers of diverse peptaibols, including trichokonins and trichorzins (Khan et al., 2020). In addition, *T. virens* synthesizes terpenoid metabolites such as viridin, which exhibit strong antifungal activity by inhibiting key enzymatic processes involved in fungal growth and metabolism (Kaur et al., 2025). Together, these compounds interfere with cellular respiration and protein synthesis, thereby enhancing antibiosis. Accordingly, the high inhibitory activity observed for *T. virens* isolates (NH7-3 and NH9-2) is consistent with their capacity to produce potent peptaibols and viridin-type metabolites. Hydrolytic enzymes present in culture filtrates further enhance chemical antagonism by degrading pathogen cell walls and increasing susceptibility to toxic metabolites. Thus, the synergism of CWDEs and secondary metabolites creates a hostile chemical environment that severely restricts *P. oryzae* growth, even in the absence of direct fungal contact.

### 5.3 Variability in the antagonistic ability of *Trichoderma* species

Across all bioassays, *Trichoderma* spp. demonstrated strong inhibition of pathogen growth, confirming the broad biocontrol potential of the genus, while notable species and mechanism-dependent variation highlight the mechanistic diversity underlying antagonistic performance.

#### 5.3.1 Species and strain-level variability

Biocontrol efficacy of *Trichoderma* is highly dependent on intraspecific variation (Singh et al., 2021). Even within a single species, strains can differ significantly in their ability to produce hydrolytic enzymes, secondary metabolites, and competition efficiency in the rhizosphere (Mathur and Mukherjee, 2025). These differences can be governed by variation in functional metabolite gene clusters (a group of closely linked genes that together are responsible for the biosynthesis of particular natural products), and differences in gene regulation and expression (Nützmann et al., 2018). Previous genomic and transcriptomic studies have demonstrated that variation in genes associated with mycoparasitism, secondary metabolite biosynthesis, and nutrient acquisition significantly influences biocontrol efficacy by shaping its metabolic arsenal, regulatory responsiveness, enzymatic capacity, competitive fitness, and interaction-specific behavior (Atanasova et al., 2013; Moisan et al., 2019). In addition, environmental stimuli can affect the production of secondary metabolites by modifying metabolic pathways, leading to changes in the synthesis of various bio compounds (Yang et al., 2018).

#### 5.3.2 Variability among antagonistic mechanisms

Distinct differences in inhibition among the evaluated antagonistic mechanisms were due to the employed mode of action of each mechanism. Dual-culture and Non-VOC bioassays consistently produced the strongest inhibition of *P. oryzae*, indicating that direct physical interaction and diffusible secondary metabolites represent the dominant mechanisms of antagonism (Garcés-Moncayo et al., 2025b). Volatile organic compounds (VOCs), although inhibitory, showed lower and more variable effects than in Non-VOC and dual-culture assays. This suggests that VOCs may play a secondary role in antagonism, potentially acting at a distance or complementing other mechanisms rather than being the primary determinant of pathogen suppression. Similar trends have been reported in other studies, where VOC-mediated inhibition was isolated and species-specific and generally less pronounced than direct, enzymatic or metabolite-mediated inhibition (Guo et al., 2019).

#### 5.3.3 Comparable performance between native *Trichoderma* spp. and commercial *Trichoderma harzianum*

Across all bioassays, native *Trichoderma* spp. consistently outperformed the commercial *T. harzianum* strain. This observation suggests that differences in antagonistic efficacy are not solely related to species identity but also influenced by strain quality and product formulation. Although commercial strains are widely used because of standardized production, their lower antagonistic performance can be associated with reduced spore viability and lower colony-forming unit (CFU) densities, which directly limit their effectiveness.

Commercial products rely on carrier materials to improve shelf life and facilitate storage and application. However, the physicochemical properties of these carriers can strongly influence propagule survival, metabolic activity, and long-term stability (De La Cruz Quiroz et al., 2019). Handling and storage may reduce spore viability and inoculum quality. Furthermore, repeated sub-culturing or long-term storage can result in genetic drift and loss of beneficial traits, making strain stability a constant concern (Mathur and Mukherjee, 2025).

In addition, the Random Forest classification model successfully integrated multiple antagonistic traits and identified elite *Trichoderma* isolates with stable, high performance across bioassays, whereby five isolates (*T. harzianum* NH3-3, *T. virens* KT8-4 and NH7-3, *T. paraviridescens* KT7-1, and T. *pleuroti* KT8-1) were the best. The emergence of these few elite isolates among many tested isolates indicates substantial functional diversity within the *Trichoderma* community, expressing stronger multi-antagonistic efficiency attributed to a combination of powerful biochemical metabolite production, physical, and vigorous mycoparasitic behavior. Elite *Trichoderma* strains are consistently identified in research through the evaluation of multiple performance criteria (Kumari et al., 2024). Superior isolates are typically characterized by high enzymatic activity, robust production of biologically active secondary metabolites, and a rapid capacity to colonize and persist in target environments. Together, these traits underpin their effectiveness as biological control agents by enhancing pathogen suppression, competitive ability, and ecological fitness.

## 6 Conclusion

*Trichoderma* spp. evaluated in this study exhibited strong, multifactorial antagonistic activity against *Pyricularia oryzae*, demonstrating that effective suppression of rice blast arises from the synergistic mode of action rather than from any single mechanism. The pronounced variability in antagonistic performance among isolates confirms that strain-level assessment is essential, as only a subset of strains consistently delivered high efficacy across assays and can therefore be considered reliable candidates for biocontrol applications. The superior and stable performance of several native isolates compared with the commercial reference strain emphasizes the value of locally adapted *Trichoderma* populations, which are likely better suited to prevailing agroecological conditions and pathogen populations, and thus represent a particularly promising resource for developing sustainable and resilient disease management strategies.

## Data Availability

The raw data supporting the conclusions of this article will be made available by the authors, without undue reservation.
